# Coping with the third wave of the COVID-19 pandemic in Africa: implications for an improved outbreak response

**DOI:** 10.2217/fvl-2021-0184

**Published:** 2021-01-17

**Authors:** Olayinka Stephen Ilesanmi, Francesco Chirico, Aanuoluwapo Adeyimika Afolabi, Gabriella Nucera

**Affiliations:** ^1^Department of Community Medicine, College of Medicine, University of Ibadan, Ibadan, Oyo State, Nigeria; ^2^Department of Community Medicine, College of Medicine, University College Hospital, Ibadan, Oyo State, Nigeria; ^3^Post-Graduate School of Occupational Health, Università Cattolica del Sacro Cuore, Roma, Italy; ^4^Department of Medicine, ASST Fatebenefratelli & Sacco. Fatebenefratelli Hospital, Emergency Unit, Milan, Italy

**Keywords:** Africa, coronavirus disease, COVID-19, health literacy, outbreak response, vaccine hesitancy

## Abstract

The third wave of the COVID-19 pandemic has commenced. To avert increase in cases and avert preventable deaths, community engagement strategies such as the promotion of vaccination, voluntary testing and debunking of COVID-19-related rumors need to be undertaken.

The novel disease COVID-19 is a pneumonia-like, respiratory infection that was initially reported from Wuhan city, China, during the winter of 2019 [1]. Due to its rapid rate of transmission, COVID-19 was declared a public health emergency of international concern on 11 January 2020 and a global pandemic on 12 March 2020 [2]. Steady rates of COVID-19 have been recorded across all the seven continents, including Asia, Europe, South America, North America, Antarctica, Oceania and Africa [1]. Africa’s index case of COVID-19 was reported in Egypt on 14 February 2020, and other countries on the continent reported their index case of COVID-19 shortly afterward [3]. In Africa the highest proportion of COVID-19 cases have been reported by South Africa, Morocco, Tunisia, Egypt, Ethiopia, Libya, Kenya, Zambia, Nigeria and Algeria [4].

Globally, the first wave of the COVID-19 pandemic commenced in October 2020. The first wave of COVID-19 peaked in January 2021 and was followed by a steady decline between January and March 2021 [5]. The second wave commenced in April 2021, peaked in May 2021 and was followed by a decline in COVID-19 cases [6]. A third wave of the COVID-19 pandemic commenced in June 2021 and is currently ongoing. As of 18 July 2021, 191,324,838 COVID-19 cases and 4,108,241 COVID-19-related deaths have been recorded [4]. In Africa, the first wave of the COVID-19 pandemic commenced in July 2020. The first wave of COVID-19 peaked in August 2020 and was followed by a steady decline in COVID-19 cases between September and November. The second wave commenced in December 2020, peaked in January 2021 and was followed by a steady decline in COVID-19 cases between February and June 2021 [5,6]. A third wave of the COVID-19 pandemic commenced at the end of June and is ongoing. As of 18 July 2021, 6,288,605 COVID-19 cases and 158,354 COVID-19-related deaths have been recorded [4].

Like the experience in other countries, many countries on the African continent adopted certain measures to contain and control the spread of the COVID-19 pandemic during the first and second waves [7]. These measures included border closure, comprehensive public health campaigns, COVID-19 testing, screening and/or quarantine for travelers, social distancing/avoidance of crowded areas and improved hygiene practices [8]. Overall, these measures contributed to keeping the COVID-19 cases at bay across the African continent [8]. Collaborative support from organizations such as the Africa CDC and other not-for-profit organizations enabled COVID-19 testing, and provision of material and financial resources required during the response activities in the first and second waves of the COVID-19 pandemic [9].

The third wave of the COVID-19 pandemic has commenced. Presently, many African countries have been caught unawares [10]. An increase in the proportion of confirmed COVID-19 cases have been reported, with many deaths occurring alongside. In many communities across the continent, it is evident that recommended safety guidelines are being disregarded, with few people wearing face masks in public places [5,10–12]. Also, in educational institutions, focal persons who enforced the use of face masks, social distancing and hand hygiene practices during the first and second waves of the COVID-19 pandemic have been found to disregard the recommended public health safety measures (PHSMs) during the third wave. If supposed enforcers of the law become the breakers of the law, how then can learners and/or teachers be encouraged to comply? To ensure that the poor health system in many African countries is not further worsened, strategies to ensure adequate response activities during the third wave of the COVID-19 pandemic should be immediately undertaken. This article; therefore, aimed to describe the strategies for ensuring adequate preparedness and response during the third wave of the COVID-19 pandemic on the African continent.

## Strategies for ensuring adequate preparedness & response during the third wave of the COVID-19 pandemic on the African continent

### Tightening of PHSMs

PHSMs such as social distancing, regular hand hygiene and compulsory use of facemasks in public places have been demonstrated as effective measures to breaking the chain of COVID-19 transmission [8]. A need for school/workplace/closure may be pertinent at certain periods to reduce the risk of exposure of many individuals to SARS-CoV-2. The aforementioned retrospective study on the effective public health safety measures during the first and second waves in Africa revealed a direct linear relationship between the stringency level and a decline in COVID-19 cases. The stringency level provided data on the tightened and loosened PHSMs during each period at national levels.

The combination of two or more PHSMs contributed to a huge decline in COVID-19 cases across the African continent [8]. Therefore, these measures should be tightened across African countries to ensure that COVID-19 cases do not rise beyond the limits that could be borne by the national health system. Due to the cost-intensive nature involved in the production of alcohol-based hand rubs and cloth face masks, partnerships should be instituted between the National Orientation Agency and private organizations in each African country. Together, these organizations could conduct skills and entrepreneurship development programs especially across both modern and traditional media on the production of alcohol-based hand rubs and reusable face masks.

### Infection prevention & control measures

Many guidelines have been developed for health facilities to ensure infection prevention and control (IPC) measures during the first and second waves of the COVID-19 pandemic [9,13,14]. These include the use of face masks and/or face shields, regular practice of handwashing, decongestion of wards and/or treatment areas and maintenance of social distancing during consultations [15]. Available evidence from many healthcare facilities in Nigeria reveals that pandemic fatigue has caused previously implemented guidelines to wane [16]. Thus, face masks and/or shields are currently supplied in inadequate quantities, thus necessitating the recycling of surgical face masks by healthcare workers. Also, unreliable water supply at many handwashing points discourages many healthcare workers and patients from observing handwashing practice.

To ensure adherence to recommended IPC measures, individual health facilities are required to conduct COVID-19 risk assessment across all the departments to rank the risks of exposure [17]. Ranking of risks will help to identify high-risk departments, such as the emergency department, to inform on the need to scale up the provision of IPC equipment and materials in such departments. In addition, a COVID-19 task force or committee should be instituted in each facility [18]. This implies that each department has a representative member in the task force or committee who is responsible for communicating the department’s IPC needs to the management and enforcing adherence to IPC guidelines. Given the unknown extent of the third and future waves of the COVID-19 pandemic, regular provision of IPC materials and equipment need to be ensured. Likewise, regular IPC training should be organized for healthcare workers to ensure that no member of the healthcare team experiences pandemic fatigue during the outbreak response in the third wave of the COVID-19 pandemic [18].

### Vaccination

Widespread acceptance of the COVID-19 vaccine is crucial to achieving total immunization coverage. To achieve total immunization coverage, vaccine hesitancy needs to be overcome among the African populace [19]. Vaccine hesitancy has been described by the WHO as the delay or blunt refusal to accept a vaccine despite the proven efficacy of the vaccine [20]. Despite the availability of the COVID-19 vaccine, vaccine hesitancy limits total coverage of the entire population in the vaccination exercise [21]. Many factors have been implicated in COVID-19 vaccine hesitancy, and these include complacency, lack of confidence in the COVID-19 vaccine stemming from misinformation, convenience and fear of side effects [21]. If not tackled immediately, vaccine hesitancy could lead to regular outbreaks of COVID-19 in African communities.

To avert such occurrences, specific COVID-19-focused actions need to be undertaken. First, community engagement through the involvement of community leaders, opinion leaders, civil-based organizations, community-based healthcare workers and teachers to dispel myths and misconceptions on the COVID-19 vaccine is urgently required [19]. Since community champions and celebrities have great influence on many individuals, endorsements and local buy-in of the COVID-19 vaccine from these community representatives need to be sought. Second, integration of the COVID-19 vaccine should be undertaken by the National Primary Healthcare Development Agency in Nigeria and other relevant agencies in other countries in Africa [19]. This will help ensure that the COVID-19 vaccine is available to all individuals who visit health facilities (primary, secondary or tertiary) for consultation or other purposes. A brief psychosocial counselling session would be undertaken by trained counsellors and/or medical social workers to ensure adequate understanding of the need for the COVID-19 vaccine, and to obtain informed consent from each person prior to vaccine administration.

### Management of infodemics

Information technology and social media have been used on a massive scale to ensure that people are kept safe and equipped with the right information to prevent COVID-19. The technology being trusted to keep the public informed amplifies an infodemic that undermines the public health response across the African continent. Owing to the novelty of the COVID-19 pandemic, a hidden epidemic of information makes COVID-19 stand out as a digital infodemic [22]. Increased levels of stress and confusion can result from repeated and detailed misinformation, rumor and conspiracy theories which increases the pollution of information regarding COVID-19. Termed ‘misinfodemic’, these arrays of information are still being spread among everyone. A network analysis on Twitter in South Africa revealed that tweets containing hashtag ‘Coronavirus’ had spread faster, and news articles containing COVID-19 were believed to be more popular [22].

To control the barrage of information circulated across the social media, it is required that recent trends in information communication technology are integrated into the package of public health information. This will help to ensure that the rights of users of modern media regarding access to adequate health information are upheld. During the second wave of the COVID-19, WhatsApp limited the sharing of messages across its platform to five individuals at a go [23]. This helped to curb the spread of false COVID-19 messages. It is required that media platforms draw evidence-based knowledge from each other to tackle the menace of COVID-19 infodemics. With the increasing use of social media platforms such as Facebook and Instagram, the Africa CDC could harness these platforms in sharing COVID-19 knowledge to improve COVID-19-related health literacy among youths and other population groups. The risk communication pillar of the African CDC and local Ministries of Health should be actively involved in debunking false COVID-19 information to promote the spread of accurate information [19]. Linkage of social media platforms to reliable sources such as the WHO, Africa CDC and Ministries of Health should be promptly executed.

### Voluntary testing

Enrolment in voluntary testing for symptomatic COVID-19 cases is a crucial step toward prompt identification of positive COVID-19 cases [24]. Evidence from Nigeria reveals that low proportions of COVID-19 testing are primarily linked to myriads of misconceptions and falsehood [25]. Misconceptions such as the use of biological samples obtained during the COVID-19 testing exercise for ulterior purposes gained acceptance during the first and second waves of the COVID-19 pandemic [19]. These misconceptions have persisted till the third wave of the COVID-19 pandemic and could deter the promising achievements associated with optimal testing. For contacts of confirmed COVID-19 cases, all persons who had been in close contact (less than 2 meters) with a confirmed case are required to enroll in voluntary testing. This will help to diagnose COVID-19-positive cases prior to the period of high infectiousness when the risk of transmissibility is at its peak [25].

To promote voluntary testing on the African continent, it is required that Africans are equipped with sufficient knowledge to promote COVID-19 health literacy [26]. In addition, COVID-19 testing centers could be decentralized to places where people naturally congregate, for example, clubs, market squares and schools [25]. However, caution needs to be taken in the dissemination of COVID-19 test results so that confirmed positive cases do not suffer stigmatization from other community members. Home-based care, a technique in which asymptomatic or mildly symptomatic COVID-19 cases are managed in their homes in separate rooms and under regular supervision of assigned healthcare workers, could be adopted for managing suspect and/or confirmed cases. Management at the isolation center is; however required for severe COVID-19 cases, especially those with underlying illnesses and complications.

Management of COVID-19 cases either in their homes or at the isolation center should be overseen by members of the COVID-19 management team [25]. In addition, people who are residents of each community should be recruited as community health volunteers [9]. The existing relationship between these volunteers and other community members could influence prompt case notification and isolation of confirmed cases. Also, this engagement could provide point-of-care testing services, and link suspected COVID-19 cases to testing. Overall, the engagement of community-based individuals will promote surveillance activities to enhance the accurate reporting of COVID-19 fatalities.

## Conclusion

The third wave of the COVID-19 pandemic has commenced across the globe and on the African continent with possibility of future waves. This therefore necessitates that swift actions are taken to ensure adequate response amid the COVID-19 pandemic. These include a scale-up and decentralization of COVID-19 testing centers. To improve individuals’ health literacy, coordinated public health campaigns focused on testing and vaccination need to be organized. These campaigns would help debunk fallacies surrounding biological samples and the COVID-19 vaccine. In addition, infection prevention and control measures and public health safety measures should be tightened in both community and healthcare settings. The engagement of community stakeholders, such as traditional and religious leaders, opinion group leaders and community-based healthcare workers will promote adequate community participation in the COVID-19 outbreak response efforts. While receiving support from other organizations, the national government in each country in Africa should build their capacity to ensure the safe integration of COVID-19 outbreak response activities into the national health system. Safe integration will ensure that these activities will be sustained long after all forms of support from supporting organizations are withdrawn.
